# *Vitis vinifera* polyphenols from seedless black fruit act synergistically to suppress hepatotoxicity by targeting necroptosis and pro-fibrotic mediators

**DOI:** 10.1038/s41598-020-59489-z

**Published:** 2020-02-12

**Authors:** Marwa M. Abu-Serie, Noha H. Habashy

**Affiliations:** 1 Department of Medical Biotechnology, Genetic Engineering, and Biotechnology Research Institute, City of Scientific Research and Technological Applications (SRTA-City), New Borg EL-Arab, 21934 Alexandria Egypt; 2 Biochemistry Department, Faculty of Science, Alexandria University, Alexandria, 21511 Egypt

**Keywords:** Target identification, Hepatotoxicity

## Abstract

Human is subjected from his surrounding to various hepatotoxins, which aggravates his liver. Nowadays, natural polyphenols have attracted great interest in health improvement, especially liver health. The present research, therefore, assessed the hepatotherapeutic potency of the isolated polyphenols (VVF1) from seedless (pulp and skin) black *Vitis vinifera* (VV) against CCl_4_-induced hepatotoxicity *in vitro* and *in vivo*. Further, VVF1 was fractionated into resveratrol-enriched (VVF2) and phenolics-enriched (VVF3) fractions to study (*in vitro*) the possible synergism of their coexistence. The highest content of phenolics in VVF1 displayed *in vitro* synergistic antioxidant and anti-hepatotoxic activities comparing to VVF2, VVF3, and silymarin (SM, reference drug). More importantly, it exhibited multiple *in vivo* regulatory functions via diminishing oxidative stress and inflammation, which in turn decreased necroptosis and pro-fibrotic mediators (mixed lineage kinase domain-like protein (MLKL), collagen type I alpha 1 chain (COL1A1), and transforming growth factor (TGF)-β1). In addition to these novel findings, VVF1 had higher anti-hepatotoxic potency than that of SM in most of the studied parameters. The histopathological analysis confirmed the improving role of VVF1 in the serious hepatic damage induced by CCl_4_. Thus, the synergistic functions of VVF1 polyphenols could be a promising new anti-hepatotoxic agent for targeting both necroptotic and profibrotic mediators.

## Introduction

The liver is the largest organ of the body and the main defense organ against environmental and metabolic toxins. Administration of certain medicinal, chemical, or industrial agents can induce hepatotoxicity. Due to the conversion of these hepatotoxins into reactive metabolites by the hepatic cytochrome P450 (CYP) metabolizing enzymes^1^. Carbon tetrachloride (CCl_4_) is a well-known hepatotoxin that caused liver cell damage by inducing hepatic oxidative stress, inflammation, and necroptosis^2,3^. Necroptosis is a pathophysiological process called programmed necrosis and combines the features of both necrosis and apoptosis-dependent inflammatory cell death. Hence, it is a highly regulated process involving specific molecules (e.g., pro-necroptotic mixed lineage kinase domain-like protein (MLKL) and at the same time associated with a membrane integrity defect^4^. All of these damage effects lead to hepatic steatosis, fibrosis, cirrhosis, and may develop into hepatocellular carcinoma if the hepatotoxin persist^2,3^.

Medicinal plants have crucial roles in health care and improving multiple human disorders. Grape (*Vitis vinifera*, VV) is one of the medicinal plants used in folk medicine and belongs to the Vitaceae family with a woody climbing vine and large leaves^5^. It can grow all over the world “non-climacteric fruit” and is considered to be the most broadly planted fruit crop and the most consumed fruits worldwide. The VV is rich in sugars, organic acids, proteins, amino acids, vitamins, minerals, and various phytochemicals including carotenoids, flavonoids, proanthocyanins, tannins, and anthocyanins. The skin of the fruit, particularly the black and red species, contains a high amount of resveratrol (stilbene derivative, polyphenolic compound), which has many health-promoting actions. VV and resveratrol have been approved for their antioxidant, anti-apoptotic, anti-carcinogenic, anti-inflammatory, immune-enhancing and neuroprotective potential^6^.

In addition, the protective roles of VV against various hepatotoxic agents such as dexamethasone^7^, paracetamol, and diethylnitrosamine were studied^8^. The hepatoprotective effects of certain parts of the fruit against CCl_4_ have also been investigated, such as roots^9^, leaves^10^, seeds^11^, and juice^12^. However, no previous studies have evaluated the therapeutic effect of the extracted polyphenols from the seedless black VV (both pulp and skin) against CCl_4_-induced hepatotoxicity in rats. Moreover, the current study evaluated the targeting ability of these extracted polyphenols to CCl_4_-induced necroptotic and pro-fibrotic mediators, which had not been previously published. These mediators (including, pro-oxidants, pro-inflammatory, pro-necroptotic MLKL protein, TGF-β and collagen type I alpha 1 chain (COL1A1) have been studied here because of their critical role in liver damage. The present study extracted the total polyphenols (fraction 1, VVF1) from the black VV (seedless fruit) using 70% (v/v) ethanol-water then separated it into ethanol-soluble components (VVF2) and water-soluble components (VVF3). The isolated fractions were studied *in vitro* for their antioxidant and anti-hepatotoxic activity to assess the probable new synergistic effect of the seedless VV polyphenols co-existence, which has not been evaluated previously. After the most effective isolated fraction was highlighted by the *in vitro* study, it was studied *in vivo* to provide more convincing evidence of its effectiveness.

## Results

### Phenolic, flavonoid, and resveratrol content of the prepared VVFs

In the present study, the VVF1, VVF2, and VVF3 were fractionated from VVCE with various extraction yields (46.76, 1.07 and 42.28 g/100 g VVCE, respectively) as elucidated in Table 1. Also, Table 1 illustrates that the most phenolics, flavonoids, and resveratrol were concentrated in VVF1 (67.62 mg 4-HCA eq, 3.901 mg RU eq, and 0.227 mg/g VVCE respectively). The ethanolic soluble fraction of VVF1 (i.e., VVF2) recorded lower phenolic and flavonoid and higher resveratrol contents than that in the water-soluble fraction of VVF1 (i.e., VVF3) as shown in Table 1. The chromatographic analysis of the highest phenolic content fraction (VVF1) showed the presence of different phenolic acids. These phenolics include gallic, vanillic, caffeic, syringic, p-coumaric, ferulic, ellagic, benzoic, o-coumaric, and salicylic according to the used polyphenolic standards (Supplementary Fig. 1).

**Table 1:** The yield and phenolic content of *Vitis vinifera* crude extract (VVCE) and its fractions (F), their safe concentrations (EC_100_) on the normal hepatocytes with their effective doses (ED) against the CCl_4_-induced *in vitro* hepatotoxicity as well as the combination index (CI) values of VVF1.

	VVCE	VVF1	VVF2	VVF3	SM
**Extracts yield and composition**					
Yield (g/100 g VVCE)	—	46.76 ± 0.065	1.066 ± 0.110***	42.276 ± 0.010	—
Phenolics (mg 4-HCA eq/g VVCE)	101.6 ± 8.674*	67.62 ± 3.303	0.444 ± 0.048**	44.35 ± 1.812*	—
Flavonoids (mg RU eq/g VVCE)	1.927 ± 0.250**	3.901 ± 0.117	0.035 ± 0.008***	4.643 ± 0.052	—
Resveratrol (mg/g VVCE)	0.031 ± 0.043*	0.227 ± 0.033	0.179 ± 0.005	0.0166 ± 0.006*	—
**Safe and therapeutic Doses**					
EC_100_ (mg/mL)	3.003 ± 0.021*	2.83 ± 0.023	1.958 ± 0.004***	2.591 ± 0.026**	2.004 ± 0.002***
ED_50_ (mg/mL)	0.400 ± 0.003**	0.161 ± 0.004	2.508 ± 0.007***	0.961 ± 0.035***	0.407 ± 0.031**
ED_100_ (mg/mL)	1.710 ± 0.086*	0.926 ± 0.024	5.689 ± 0.155***	2.124 ± 0.091**	1.952 ± 0.040**
**CI value for VVF1 at**	**β-carotene–linoleate bleaching**		**DPPH**	**Superoxide radical**	**Hydroxyl radical**
50% Inhibition	0.110 ± 0.004		1.72 × 10^−5^ ± 0.000	0.085 ± 0.000	0.273 ± 0.001
75% Inhibition	0.206 ± 0.000		1.26 × 10^−7^ ± 0.000	0.038 ± 0.001	0.221 ± 0.007
90% Inhibition	0.387 ± 0.005		9.7 × 10^−10^ ± 0.000	0.021 ± 0.000	0.180 ± 0.008

Results are presented as mean ± SE (n = 3). VVF1 was compared with VVCE, VVF2, VVF3 and SM and considered significantly different at *p < 0.05, **p < 0.005, ***p < 0.0005. 4-HCA, 4-hydroxycinnamic acid; RU, Rutin; SM, Silymarin; ED_50_ and ED_100,_ 50% and 100% therapeutic response against the CCl_4_-induced *in vitro* hepatotoxicity, respectively; DPPH, 2,2-diphenyl-1-picrylhydrazyl.

### Antioxidant activities of VVFs

**Figure 1:**
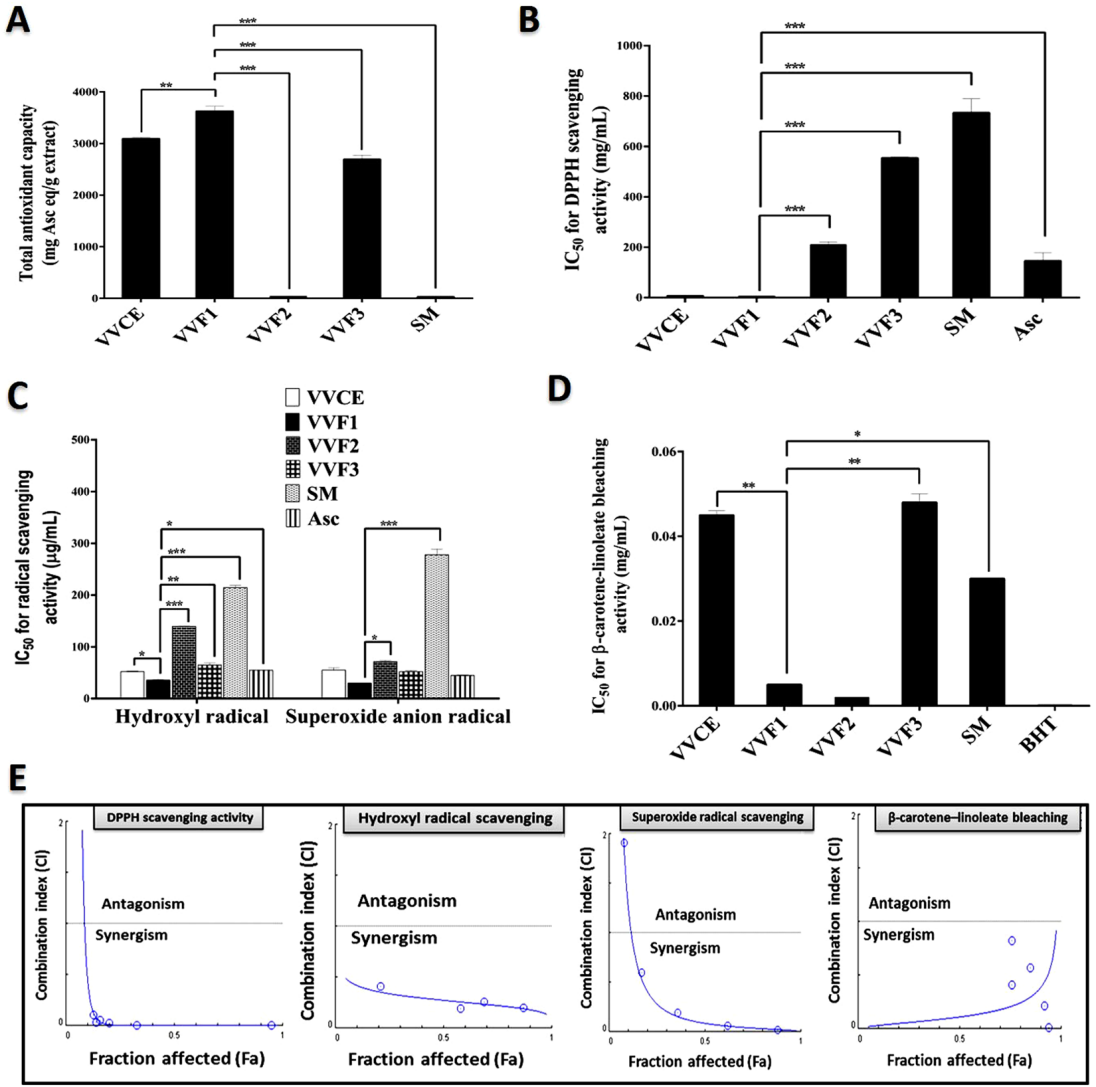
Antioxidant content and efficacy of VVF1 with a combination index (CI) plot of its constituents for radical scavenging and anti-lipid peroxidation activities. (**A**) Total antioxidant capacity (TAC), (**B**) DPPH scavenging activity, (**C**) hydroxyl and superoxide anion radical scavenging activities as well as (**D**) anti-lipid peroxidation activity of VV crude extract (VVCE), VVF1 and its fractions (VVF2 and VVF3) in comparison with silymarin (SM), Asc and butylated hydroxytoluene (BHT). (**E**) CI graph of antioxidant synergism of VVF2 and VVF3 (VVF1’s constituents) including, DPPH, hydroxyl, and superoxide radical scavenging activities and inhibitory activity of lipid peroxidation. VVF1 was compared with VVCE, VVF2, VVF3, SM, Asc, and BHT and considered significantly different at *p < 0.05, **p < 0.005, ***p < 0.0005.

Figure 1A illustrates that VVF1 had the highest total antioxidant capacity (3624 mg ascorbic acid “Asc” eq/g extract) in comparison with VVCE, other VVFs, and silymarin (SM; a standard drug for hepatotoxicity). Based on low IC_50_ indicating high antioxidant activity, VVF1 revealed the strongest radical scavenger for DDPH, hydroxyl and superoxide anion radicals at the lowest IC_50_ (3.6, 35.78 and 30.07 µg/mL, respectively) compared to other VVFs. The radical scavenging activity of VVF1 was not statistically significant with that of VVCE. Also, no significant difference was recorded between VVF1 and Asc (antioxidant marker) for hydroxyl and superoxide anion radical scavenging activity. Regarding DPPH scavenging potential, VVF1 was significantly (p < 0.0005) higher than Asc (Fig. 1B,C). Moreover, VVF1 exhibited the highest anti-lipid peroxidation activity by preventing linoleate oxidation then bleaching β-carotene at the lowest IC_50_ (0.005 mg/mL). This value was not statistically significant with BHT (standard antioxidant) and VVF2 as shown in Fig. 1D.

The synergism between VVF2 and VVF3 for the studied antioxidant activities was declared by different methods, the CI values at different inhibition levels (50%, 75%, 90%, Table 1), Fa-CI plot (Fig. 1E), as well as isobologram plot (Supplementary Fig. 2A–D). The values of CI were lower than 1 at the studied inhibition levels for all the tested antioxidant assays. Isobolograms were performed, in particular at 50%, 75%, and 90% inhibition levels of Fa. Hence the single-agent Fa value corresponds in our study to the IC_50_ value, the isobologram at 50% inhibition for the combination provided a direct comparison with single-agent treatment and synergy refers to a lowering of IC_50_ equivalent (left-shift). While 75% and 90% isobologram refers to the combination at a high effect level. The findings of both the Fa-CI and the isobologram plots showed that the combination of VVF2 and VVF3 had strongly synergistic anti-DDPH activity and that the least amount of synergism was observed with the hydroxyl radical. This antioxidant synergism between water-soluble polyphenolic and ethanol-soluble polyphenolic constituents of VVF1 resulted in its powerful antioxidant efficacy (Fig. 1A–E).

### The antihepatotoxicity potency of VVF1 among VV fractions (*in vitro*)

**Figure 2:**
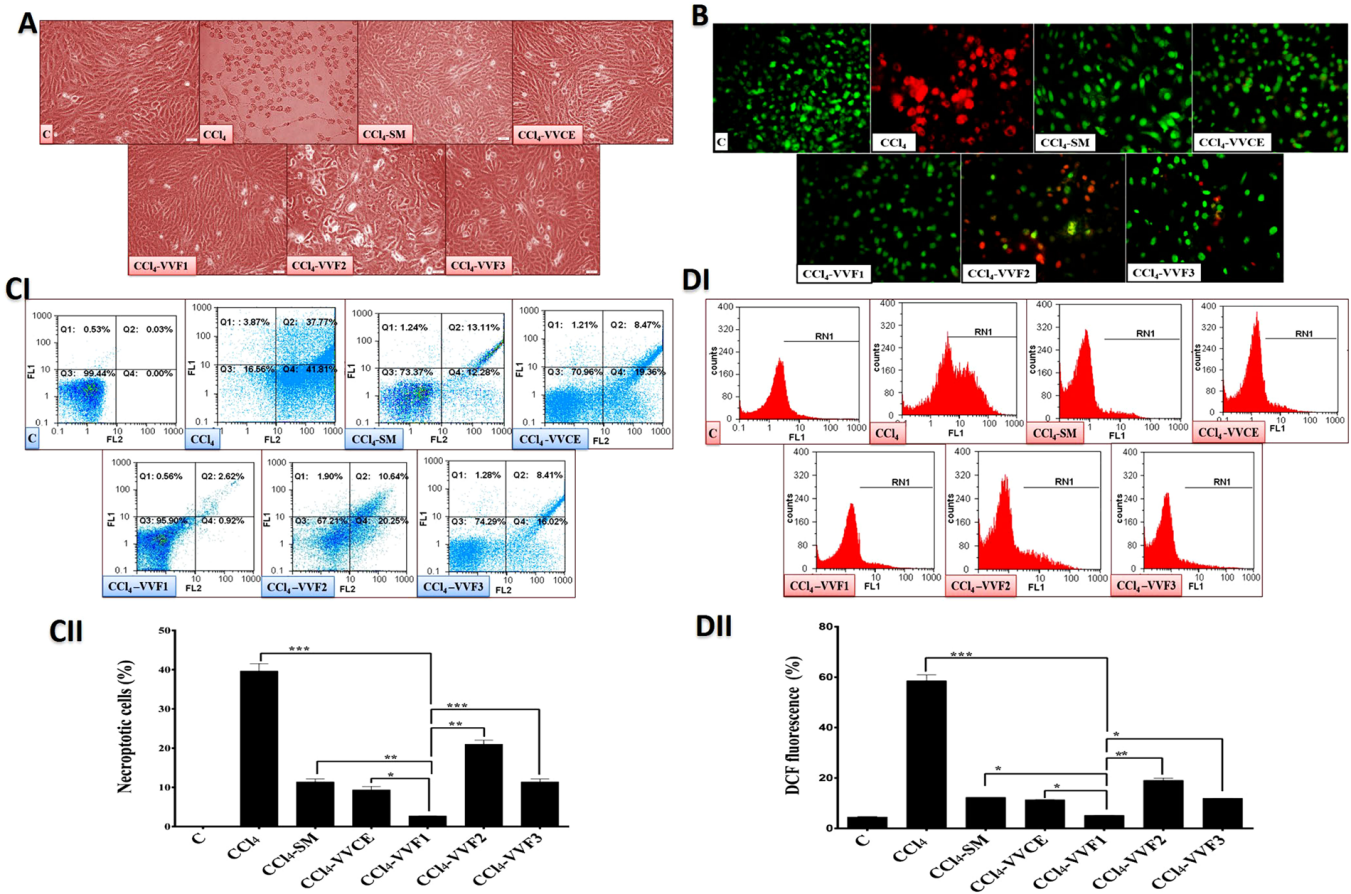
Morphological and flow cytometric analyses of the anti-necroptotic potency of VVF1. (**A**) The phase-contrast microscopic images of necrotic hepatocytes exposed to CCl_4_ without any following treatment in comparison with the SM-, VVCE- and its fraction-treated hepatocytes which previously incubated with CCl_4_. (**B**) Fluorescence images of acridine orange and ethidium bromide nuclear staining of CCl_4_-exposed hepatocytes before and after treatment with VVCE and its fractions as well as SM. Green, yellow and orange-red fluorescences refer to healthy alive, early necroptotic and late necroptotic cells. (CI,II) Annexin V/PI flow charts with quantification histogram of the percentages of double-positive Annexin V/PI-stained necroptotic and (DI,II) flow cytometric charts of fluorescence oxidized form of DCF diacetate (indicator of reactive oxygen species “ROS”) with quantitative histogram for the percentage of intracellular ROS for the untreated and treated necrotic hepatocytes. C, control untreated healthy hepatocytes; CCl_4_, hepatocytes were exposed to CCl_4_ without any treatment; CCl_4_-SM, CCl_4_-VVCE, CCl_4_-VVF1, CCl_4_-VVF2, and CCl_4_-VVF3 hepatocytes were exposed for 36 h to CCl_4_ then treated for 72 h with SM, VVCE, VVF1, VVF2 and VVF3, respectively. Data are presented as Mean ± SE. CCl_4_-VVF1 was compared with C, CCl_4_, V, and CCl_4_-SM and considered significantly different at *p < 0.05, **p < 0.005, ***p < 0.0005.

Table 1 illustrates that the safe doses (EC_100_) of VVCE and VVFs were 2 to 3 mg/mL on rat hepatocytes. These doses were used to investigate the therapeutic effect of VVFs on CCl_4_-induced hepatotoxicity in comparison with the standard drug (SM). The treatment of the CCl_4_-exposed hepatocytes with VVCE and its fractions showed that VVF1 diminished hepatotoxicity by 50% at the lowest dose (ED_50_ = 0.161 mg/mL) compared to ≥0.4 mg/mL in case of VVCE, other tested VVFs, and SM. Further, VVF1 had the lowest estimated therapeutic dose (ED_100_ = 0.926 mg/mL) for complete inhibition of hepatotoxicity while it was needed above 1.7 mg/mL of VVCE, other tested VVFs or SM for reaching the same effect using MTT assay (Table 1). Additionally, the treatment of CCl_4_-exposed hepatocytes with VVF1 was able to maintain normal morphology of hepatocytes which was near to that of healthy untreated control cells (Fig. 2A). Meanwhile, CCl_4_-exposed hepatocytes had severe damage in their spindle shape (cell rounding with cytoplasmic swelling). This indicated the incidence of necrosis that was also assured by the red fluorescence of their nuclei in contrast to green fluorescence nuclei of healthy control cells after incubation with dual nuclear staining of acridine orange and ethidium bromide (Fig. 2B). The 72 h treatment with VVF1 or VVCE did not show any red swollen nuclei, referring to halt necroptosis. Whereas the treatment of CCl_4_-exposed hepatocytes with other VVFs or SM still had few reddish or yellowish-orange nuclei of late or early necroptotic cells, respectively (Fig. 2B).

For a more accurate estimation of the anti-necroptotic effect of VVF1, the treated hepatocytes were stained with annexin-propidium iodide (PI) then analyzed using flow cytometry for detecting the percentage of double-positive annexin-PI stained cell populations. Figure 2C(I,II) shows that 39.64% of CCl_4_-exposed hepatocytes were necroptotic population. Hence, the induction of necroptosis in CCl_4_-exposed hepatocytes was evidenced by an increase in cell size (Fig. 2A,B) and an abnormally high percentage of double-positive annexin-PI stained cells. Also, Fig. 2C(I,II) illustrates that the lowest percentage (p < 0.0005) of necroptotic populations (2.68 ± 0.06%) were recorded in VVF1-treated CCl_4_-exposed hepatocytes in comparison with 9.35–20.99% in VVCE, VVF2, VVF3, and SM. The high necroptotic percentage in CCl_4_-exposed hepatocytes was clarified by elevating the percentage of the dichlorofluorescein (DCF) fluorescence from 4.519 ± 0.174% of healthy control cells to be 58.55 ± 2.28% after 72 h of CCl_4_ exposure (Fig. 2DI,II). VVF1 exhibited the highest efficiency to reduce this percentage that reflected the generation of reactive oxygen species (ROS) to about 5% in comparison with VVCE, VVF2, VVF3, and SM (11.32, 19.065, 10.915, and 11.91%, respectively).

Moreover, the estimated CI of VVF1 for blocking the induction of necroptosis and generation of the fluorescent DCF was <1 (0.166 ± 0.005 and 0.479 ± 0.02, respectively) confirming the high synergism between VVF2 and VVF3 constituents of VVF1.

### The therapeutic effect of VVF1 on CCl_4_-induced hepatotoxicity (*in vivo*)

**Figure 3:**
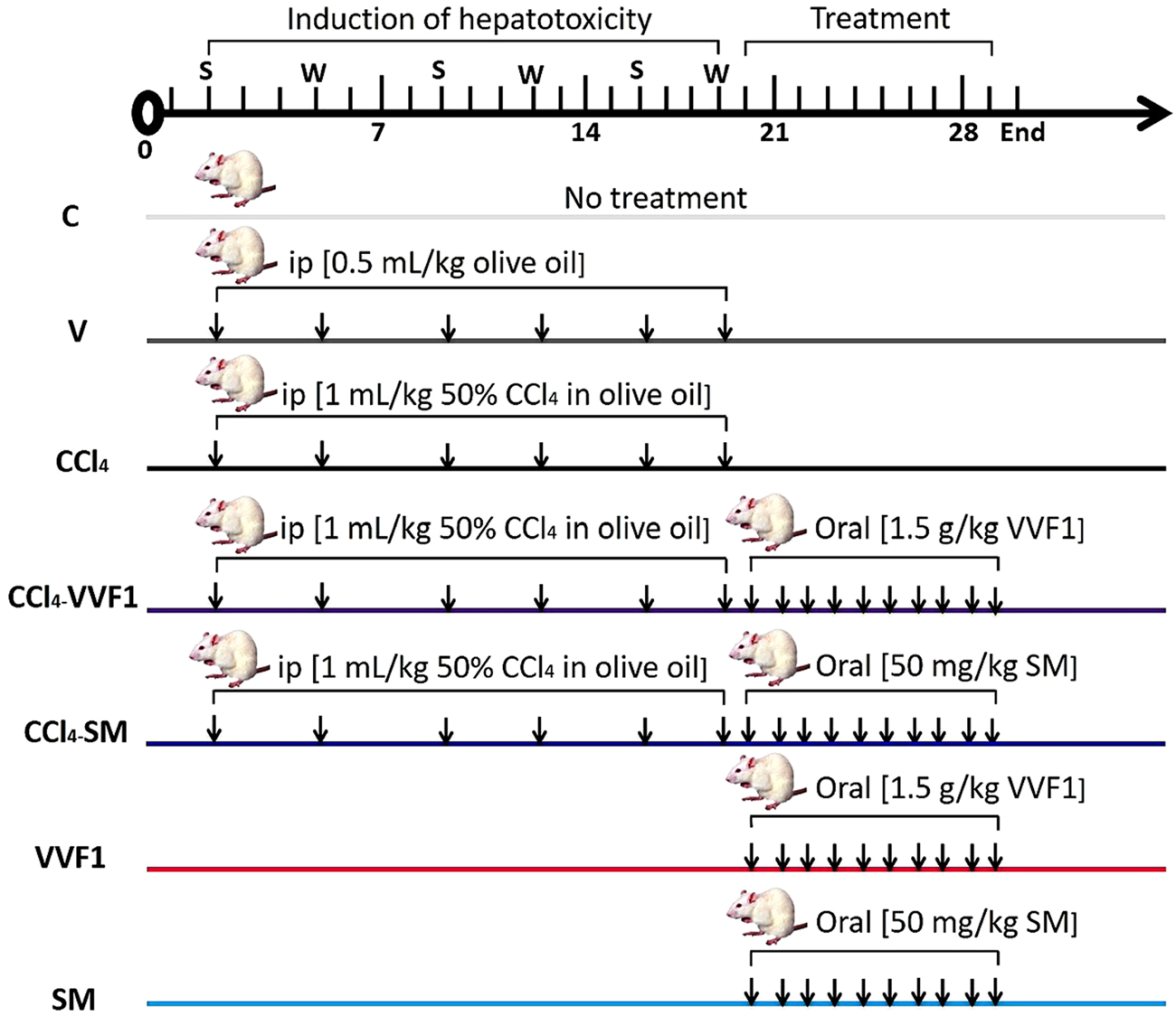
Experimental design with animal group classification. Induction of hepatotoxicity was done by injection CCl_4_ group with CCl_4_ twice (Sunday “S” and Wednesday “W”)/week for 3 weeks. After induction of hepatotoxicity, CCl_4_-VVF1 and CCl_4_-SM rat groups were treated with VVF1 and SM, respectively for 10 days. Two healthy rat groups (VVF1 and SM) were orally injected with VVF1 and SM daily for 10 days in comparison with control untreated healthy group (C) and another group (V) were injected with the vehicle of CCl_4_ (olive oil) twice/week for 3 weeks.

Based on the above-mentioned *in vitro* results, VVF1 was selected to investigate its anti-hepatotoxicity efficacy using an animal model. The induction of hepatotoxicity using CCl_4_ was followed by treatment with VVF1 (CCl_4_-VVF1 group) comparing with the standard drug (CCl_4_-SM group) as elucidated in Fig. 3.

### Returning the hepatic redox stress balance and suppression of necroptotic and fibrotic driving forces by VVF1

**Figure 4:**
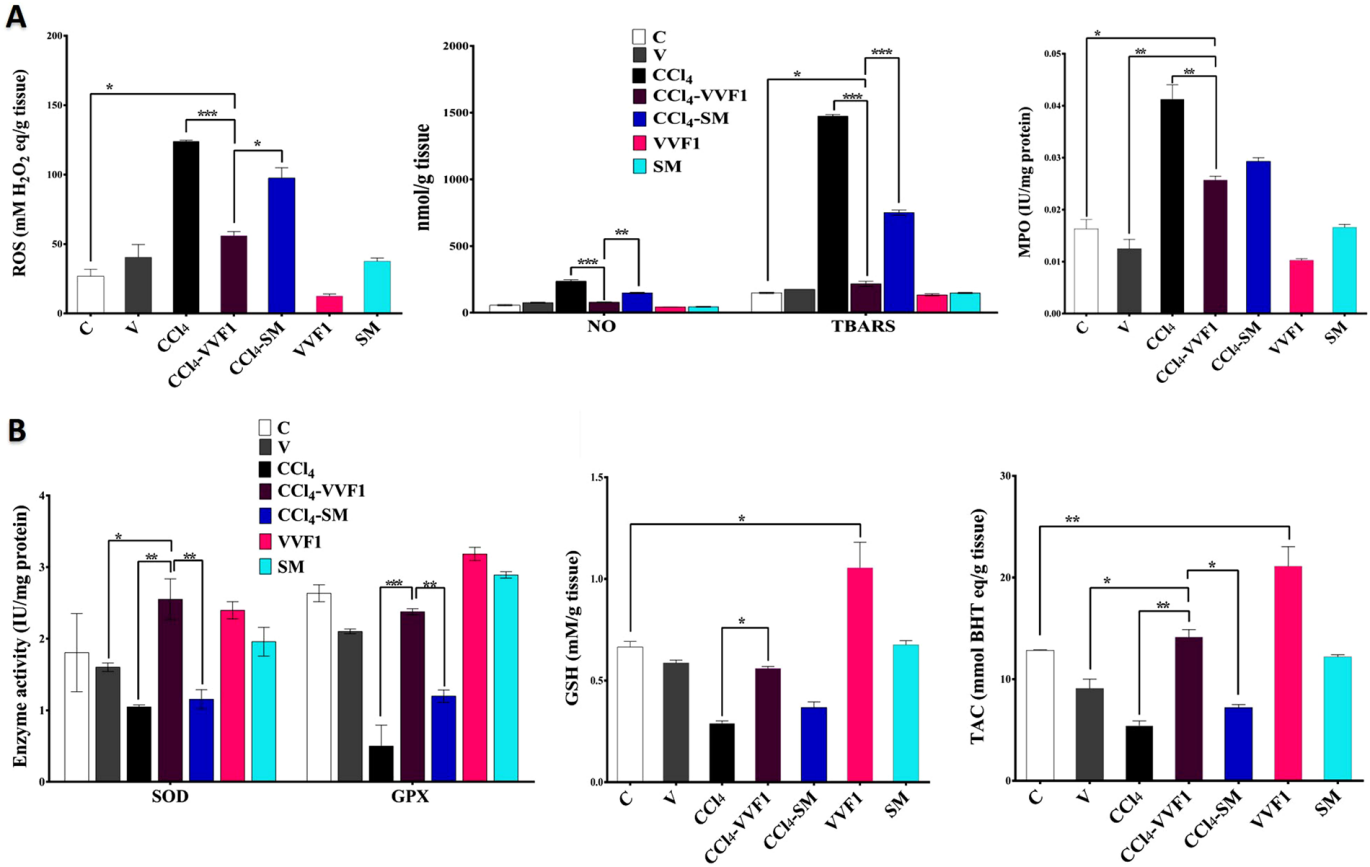
*In vivo* antioxidant- and anti-inflammatory dependent anti-necroinflammatory potency of VVF1. (**A**) Hepatic contents of ROS, NO and lipid peroxide product (TBARS) as well as hepatic activity of myeloperoxidase (MPO) in all animal groups. (**B**) Hepatic activities of superoxide oxide dismutase (SOD) and glutathione peroxidase (GPX) as well as the level of the reducing form of glutathione and TAC in all experimental rat groups. (**C**) Control untreated healthy rat group; V, olive oil (vehicle of CCl_4_)-injected rats; CCl_4_, CCl_4_-induced hepatotoxicity group; CCl_4_-VVF1 and CCl_4_-SM; the treatment of CCl_4_-induced hepatotoxicity with VVF1 and SM, respectively; VVF1 and SM, healthy rats were treated with VVF1 and SM, respectively. CCl_4_-VVF1 was compared with C, CCl_4_, V, and CCl_4_-SM while VVF1 was compared with C and SM. These comparisons were considered significantly different at *p < 0.05, **p < 0.005, ***p < 0.0005.

Oxidative stress and inflammation are cross-linked driving forces of necroptosis-dependent hepatotoxicity. Figure 4A shows CCl_4_ induced hepatic oxidative stress that was clarified by the significant elevation of ROS (124.18 ± 0.628 mM H_2_O_2_ eq/g tissue) and NO (236.54 ± 10.79 nmol/g tissue) leading to an excess generation of lipid peroxide products (1473.4 ± 12.50 nmol/g). This is associated with 2 folds enhancement of myeloperoxidase (MPO) activity as well as suppression of enzymatic antioxidant activities (superoxide dismutase “SOD” and glutathione peroxidase “GPX”) and the GSH level by 1.7, 5.3 and 2.3 folds, respectively compared to the control (C) (Fig. 4B). Thus, hepatic total antioxidant capacity (TAC) was significantly lowered in the CCl_4_-injected rat (CCl_4_ group) by 3 folds than C group. Meanwhile, the treatment of CCl_4_-injected rat group with VVF1 (CCl_4_-VVF1 group) diminished the prooxidant parameters (p < 0.0005), including ROS, NO, TBARS and MPO activity by 54.91%, 66.58%, 85.29%, and 37.77%, respectively, compared to the CCl_4_ group (Fig. 4A). Furthermore, VVF1 was able to enhance the hepatic antioxidant system (SOD, GPX, and GSH) by ≥2 folds that also corroborated by 2.6 folds increase in the TAC level relative to the CCl_4_ group (Fig. 4B). VVF1 was significantly exhibited a higher potency to halt ROS, NO, and TBARS production and to ameliorate SOD and GPX as well as TAC than standard drug (SM). However, there was no significant difference was observed between the effect of VVF1 and SM on MPO and GSH. On the other hand, injection with a CCl_4_ vehicle (V group) caused non-significant alteration of the oxidative stress parameters compared to the control rats. Regarding the change in liver weight relative to body weight (b.w.), there was no significant difference was recorded between all the studied groups, data not shown.

**Figure 5:**
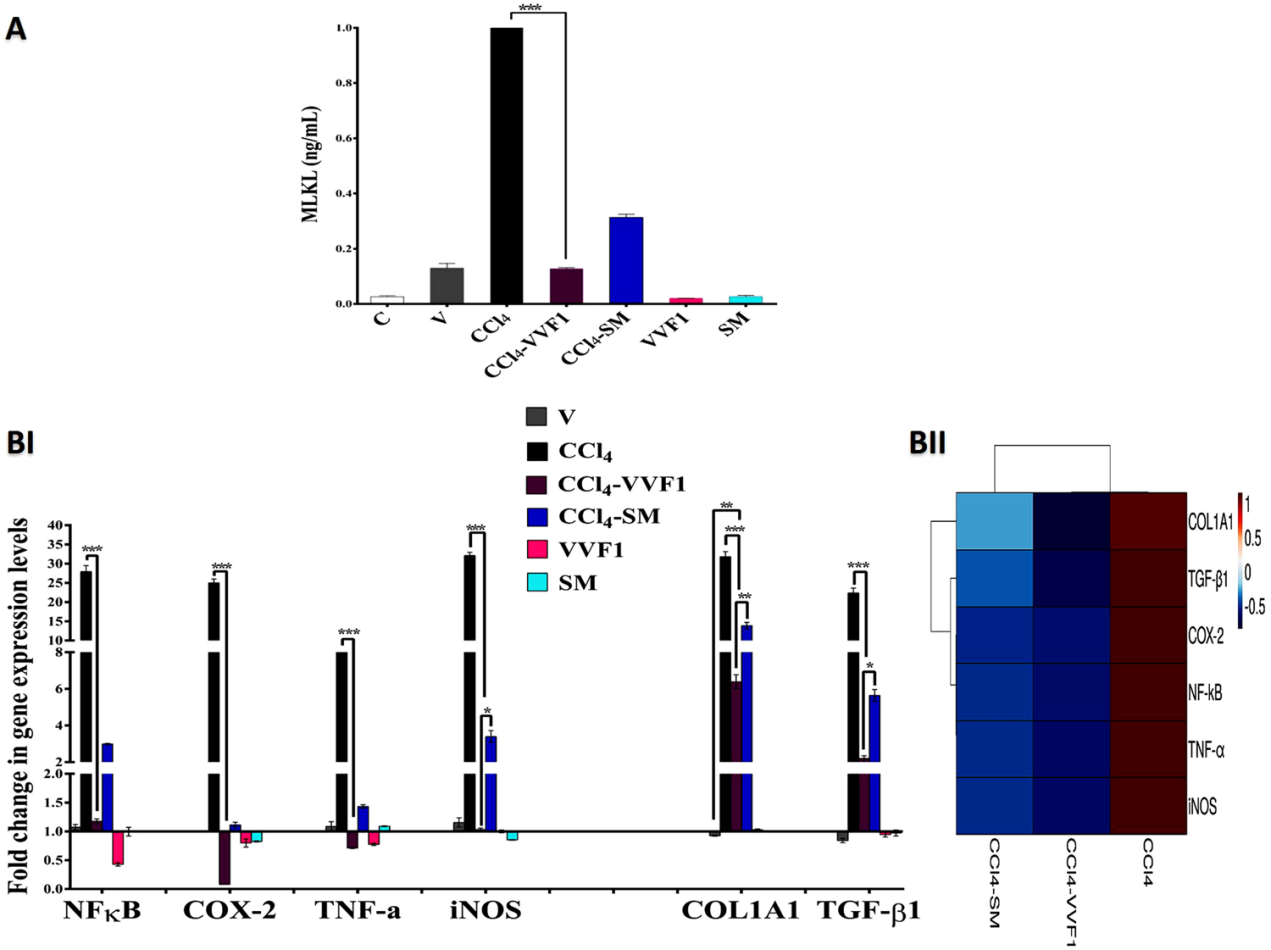
Inhibitory effect of VVF1 on pronecroptotic protein and expression of some key genes. (**A**) The pro-necroptotic protein level of mixed lineage kinase domain-like protein (MLKL) and (BI,II) the fold change in gene expressions of NF-kB, cyclooxygenase (COX)-2, tumor necrosis factor (TNF)-α, inducible nitric oxide synthase (iNOS), collagen type I alpha 1 chain (COL1A1) and transforming growth factor (TGF)-β1 in rat liver tissues with its heat map distribution that represents the relative gene expressions of CCl_4_, CCl_4_-VVF1 and CCl_4_-SM groups, the color distributed from blue (downregulated genes) to red (upregulated genes). C, control untreated healthy rat group; V, olive oil (vehicle of CCl_4_)-injected rats; CCl_4_, CCl_4_-induced hepatotoxicity group; CCl_4_-VVF1 and CCl_4_-SM; the treatment of CCl_4_-induced hepatotoxicity with VVF1 and SM, respectively; VVF1 and SM, healthy rats were treated with VVF1 and SM, respectively. CCl_4_-VVF1 was compared with C, CCl_4_, V, and CCl_4_-SM while VVF1 was compared with C and SM. These comparisons were considered significantly different at *p < 0.05, **p < 0.005, ***p < 0.0005.

Figure 5A illustrates that CCl_4_ significantly upregulated the necroptotic protein MLKL by 92.6 folds comparing to C group while this elevation was repressed to 4.85 and 11.87 folds after the treatment with VVF1 and SM, respectively. This result suggested that VVF1 has shown a higher efficacy in alleviating the elevation in MLKL than SM.

Some key genes including nuclear factor-kappa (NF-к)B, cyclooxygenase (COX) 2, tumor necrosis factor (TNF)-α, inducible nitric oxide synthase (iNOS), collagen type I alpha 1 chain (COL1A1), and transforming growth factor (TGF)-β1 were investigated. As shown in Fig. 5B, all the studied mediators of necroinflammation (NF-кB, COX-2, TNF-α, and iNOS) and fibrosis (COL1A1 and TGF-β1), at the gene expression level, were upregulated by 9–32.11 folds in CCl_4_ group relative to C group. VVF1 was able to significantly downregulate the gene expression (p < 0.0005) of all the above-mentioned markers by 1.177, 0.0798, 0.7122, 1.031, 6.388 and 2.202 folds, respectively. Whereas, the treatment of CCl_4_-injected rats with SM (CCl_4_-SM group) decreased the relative expression of these genes by 2.99, 1.115, 1.433, 3.395, 13.863 and 5.649 folds, respectively. The efficiency of VVF1 in suppressing mRNA levels of these markers, particularly profibrotic factors, was more potent (p < 0.05) than SM (Fig. 5BI). Additionally, the intensities of all studied gene expression, for CCl_4_, CCl_4_-VVF1, and CCl_4_-SM, were illustrated by a heat map with a color change from blue (downregulating gene) to red (upregulating gene) as shown in Fig. 5BII.

The injection of VVF1 or SM to healthy rats (VVF1 group or SM group, respectively) did not cause any abnormal changes in the hepatic redox status or any elevation in the parameters of inflammation, necroptosis or fibrosis. More interestingly, the injection of healthy rats with VVF1 resulted in a significant increase in hepatic GSH in the C group (Figs. 4 and 5). In this study, it was noted that the injection of rats with a CCl_4_ vehicle (olive oil, V group) did not show a strong effect on the most tested parameters. This declares that the hepatotoxic effect of CCl_4_ was mostly attributed to its potency alone (Figs. 4 and 5).

### Preserving liver morphology, architecture, and functions in CCl_4_-VVF1 group

**Figure 6:**
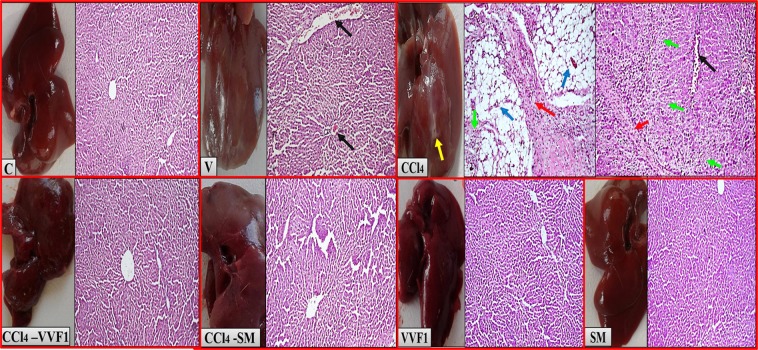
Liver morphology and histopathological images confirming the therapeutic potency of VVF1 as an anti-hepatotoxic agent. C, VVF1 and SM groups showing healthy liver (reddish-brown liver color). Congestion (black arrow) in V group and necrosis (green arrows) with dense fibrous bands (red arrows) as well as steatosis with clusters of inflammatory cells (blue arrows) in the CCl_4_ group (brown liver color with the abnormal white color area “yellow arrow”). No necroinflammation, steatosis, and fibrosis were shown in CCl_4_-VVF1 and CCl_4_-SM (with dilated sinusoids), magnification x200. C, control untreated healthy rat group; V, olive oil (vehicle of CCl_4_)-injected rats; CCl_4_, CCl_4_-induced hepatotoxicity group; CCl_4_-VVF1 and CCl_4_-SM; the treatment of CCl_4_-induced hepatotoxicity with VVF1 and SM, respectively; VVF1 and SM, healthy rats were treated with VVF1 and SM, respectively.

Histopathological analysis of rat livers (brown color liver) of CCl_4_ group confirmed the incidence of necroinflammation with dense fibrosis bands that accompanied with steatosis (accumulation of fat vacuoles) comparing to only congestion in case of V group. Meanwhile, liver tissues of CCl_4_-VVF1 group, VVF1 group or SM group showed normal liver image (dark reddish-brown color) and healthy hepatocytes like C group with no signs of necroinflammation, steatohepatitis or fibrosis. Also, the CCl_4_-SM group had normal liver (slight reddish-brown color) but with dilated sinusoids (Fig. 6).

Additionally, Table 2 illustrates that the CCl_4_ group exhibited a defect in liver function which was indicated by lowering blood albumin and increasing blood cholesterol compared to C group. However, treatment with VVF1 normalized blood levels of albumin and cholesterol. This enhancing effect of VVF1 on liver function was higher than that of SM (p > 0.05). There was no significant difference between all experimental groups regarding alanine aminotransferase (ALT) and aspartate aminotransferase (AST) activity (Table 2).

**Table 2:** Liver function markers and cholesterol level in the sera of rats in all the studied groups.

Groups	Albumin (g/dL)	ALT (IU/mL)	AST (IU/mL)	Cholesterol (mg/dL)
C	4.272 ± 0.166	43.167 ± 6.641	872.000 + 69.637	23.455 ± 2.052
V	3.742 ± 0.281	64.556 + 3.731	847.290 ± 94.022	13.098 ± 2.510
CCl4	2.926 ± 0.180**	27.700 ± 0.583	808.140 ± 96.285	*51.832 ± 15.89
CCl4-VVF1	4.492 ± 0.406	50.538 ± 0.700	842.733 ± 60.505	35.776 ± 2.863
CCl4-SM	3.222 ± 0.194	1.8336 ± 4.879	862.333 ± 157.410	26.701 ± 7.617
VVF1	4.077 ± 0.277	44.214 ± 1.364	832.551 ± 48.791	20.535 ± 5.368
SM	3.930 ± 0.256	44.901 ± 2.227	689.182 ± 53.572	8.289 ± 1.569

Results are presented as Mean ± SE (n = 7). CCl_4_-VVF1 was compared with C, CCl_4_, V, and CCl_4_-SM while VVF1 was compared with C and SM. These comparisons were considered significantly different at *p < 0.05, **p < 0.005, ***p < 0.0005. *ALT*, alanine aminotransferase*; AST*, aspartate aminotransferase; *SM*, Silymarin; *VVF1*, *Vitis vinifera* fraction 1; *V*, vehicle.

## Discussion

Herbal products have been used as drugs for thousands of years and recorded from many ancient cultures as historical evidence. VV is one of those herbs that are rich in phenolic compounds with other constituents. We prepared three different phenolic fractions from the black VV seedless fruit (pulp and skin) crude extract (VVCE), VVF1, VVF2, and VVF3. The results found that VVF1, which represents the highest yield, had the greatest phenolic content compared to VVF2 and VVF3. Phenolic compounds are widely known for their successful antioxidant activities through different mechanisms such as electron or hydrogen donors to prevent the chain radical reaction^13^. Therefore, VVF1, among the prepared fractions of VV, had the strongest antioxidant activities and was able to scavenge different types of free radicals as shown in Fig. 1. Moreover, VVF1 showed greater antioxidant activity than BHT or Asc and also SM. In addition, VVF1 exhibited synergistic antiradical activities against hydroxyl, superoxide, and DPPH radicals as well as anti-lipid peroxidation (β-carotene-linoleate bleaching inhibition activity). This synergistic antioxidant effect of VV polyphenols has mediated the powerful efficiency of VVF1 among VVFs. The HPLC analysis revealed different types of phenolic compounds in VVF1, some of which demonstrated their synergism due to their coexistence or combination. The previous studies revealed synergistic antioxidant activities of mixing some phenolic compounds such as vanillic, gallic, and chlorogenic acids^14^ as well as catechin and resveratrol^15^. Furthermore, the combination of rosmarinic acid and quercetin, rosmarinic acid and caffeic acid and the mixture of caffeic acid, ferulic acid, and epigallocatechin-3-gallate demonstrated synergistic antioxidant actions^16^. VVF1 showed also more potent antioxidant activities than the crude extract (VVCE). This may be due to the enrichment of this fraction mainly with phenolic compounds and/or the presence of certain antagonistic interactions between different constituents in VVCE. Hence, certain previous studies found that the combination of caffeic acid and α-tocopherol exerted antagonistic antioxidant effect^15^.

The present study was *in vitro* evaluated the potential role of the three VV prepared fractions against CCl_4_-induced hepatotoxicity. The CCl_4_ is a well-known hepatotoxin that caused hepatic oxidative stress, inflammation, necroptosis, fibrosis as well as liver cancer in rats^17^. CCl_4_ metabolizes by the hepatic Cytochrome P4502E1 (CYP2E1) producing trichloromethyl free radical (CCl3^*^) that combines with oxygen to generate the more reactive trichloromethyl peroxyl radical (CCl3OO^*^)^18^. Therefore, the level of ROS was elevated in the hepatocytes after their exposure to CCl_4_ (Fig. 2DI,II) that resulted in oxidative damage-dependent necroptosis promoting liver injury. The latter was assured by the increasing size of hepatocytes and the loss of their normal spindle shape as well as red fluorescence of AO/EB-stained swollen nuclei with a high percentage of annexin-PI stained necroptotic cells (39.64%) as shown in Fig. 2A–D.

The treatment with SM or VVFs clearly improved this damage as appeared under the light and fluorescence microscope (Fig. 2A,B). Moreover, VVF1 showed the most potent efficacy in suppressing the oxidant-mediated necroptosis compared to the other two fractions (p < 0.005), crude extract (p < 0.05), and SM (p < 0.005). This may be due to its strongest antiradical activity (Fig. 1) that enable it to scavenge the most generated free radicals of CCl_4_ metabolism in hepatocytes compared to other VVFS and SM (Fig. 2D) and in turn, prohibited the necroptosis-dependent cellular damage. These findings were consistent with the previous studies that proved the significance of phenolic-rich extracts in avoiding CCl_4_-induced hepatic damage^19^. Furthermore, VVF1 exhibited a synergistic (CI ˂ 1) effect in lowering the ROS level and necrotic hepatocytes, which may be related to the synergistic antioxidant action of its phenolic contents as discussed above.

The current study also investigated the therapeutic effect of the most potent VV fractions (VVF1) on the CCl_4_-induced hepatotoxicity in rats to confirm the *in vitro* outcomes. The injection of CCl_4_ in rats resulted in ROS production in liver tissue, which deactivated with the cellular antioxidants to maintain its threshold level. After multiple injections of CCl_4_, the elevated ROS level, altering the redox state, and damaging the cellular macromolecules were observed. The membrane lipids are the most susceptible to ROS damage causing lipid peroxidation that mediated cellular oxidative damage. The antioxidant defense system including GSH, SOD, and GPX was depleted after raising lipid peroxidation and ROS^20^ causing a reduction in the hepatic TAC level. This imbalance between the ROS level and the antioxidant defense system has led to oxidative stress conditions in the hepatic tissue. The present findings were in accordance with the previous study of Adewale *et al*.^21^.

The treatment of CCl_4_-administered rats with VVF1 restored the oxidative damage by diminishing the ROS level, inhibiting lipid peroxidation as well as normalizing the hepatic antioxidant indices and TAC (Fig. 4). These results were in harmony with our *in vitro* outcomes, which elucidated the potential role of VVF1 in quenching superoxide, hydroxide, and peroxide radicals. This may be owed to the phenolic content of VVF1, which not only scavenged the generated ROS from CCl_4_ metabolism but also improved the antioxidant defense system. The enhancing antioxidant abilities of gallic^22^, vanillic^23^, caffeic, syringic, p-coumaric, ferulic, ellagic, and salicylic acids^24^, as well as flavonoids^25^, have been reported previously. In addition, the administration of VVF1 alone without CCl_4_ improved the redox state (TAC) of the liver tissue by a significant increase in the level of GSH. This may be due to certain constituents in this fraction, which are capable of increasing the expression of γ-glutamylcysteine synthetase, the rate-limiting enzyme of GSH synthesis, such as flavonoids^25^. Increasing the level of GSH will result in increasing GPX activity (p > 0.05) due to its essential role as enzyme co-substrate^20^. These results are in line with the previous work of Ragab *et al*. who proved the role of VV seed extract in improving the CCl_4_-induced oxidative stress in rat liver^11^. The current study found an improvement in the antioxidant status of the liver after treatment with SM, but with extremely less potency than VVF1. The antioxidant mechanism of SM was known before and related to its direct ROS scavenging ability, inhibiting ROS-producing enzymes, activating enzymatic and non-enzymatic antioxidants^26^. The ability of SM to overcome the CCl_4_-induced oxidative damage in the liver was reported previously^27,28^. Moreover, the present study found that SM had no significant effect on the antioxidant status of the liver tissue upon its oral administration to normal rats for ten days.

The present research assessed the anti-inflammatory function of VVF1 in CCl_4_-induced hepatic necroptosis in addition to the oxidative stress. Hence, there is strong crosstalk between these two damage effects for the induction of necroptosis-dependent hepatotoxicity. Here, we evaluated certain prooxidant inflammatory parameters, including NF-κB, COX-2, TNF-α, iNOS, NO, and MPO, all of which were raised after CCl_4_ injection. ROS generation could modulate the NF-κB response and its target genes, including COX-2, TNF-α, and iNOS. The latter is responsible for the formation of NO that can interact with superoxide radical resulting in the creation of extremely reactive peroxynitrite. However, COX-2 catalyzes the conversion of arachidonic acid to prostaglandin H2 in addition to the generation of superoxide radicals as side products. All of these mediators, alongside TNF-α, which amplify NF-кB, have potentiated CCl_4_ damage in hepatic tissue^29^. In addition, CCl_4_ was correlated with an increase in MPO activity which magnifies the inflammatory signal and stimulates lipid peroxidation in the presence of halide ions and H_2_O_2_^30^. This may be related to its principal role in the formation of hypochlorous acid (HOCl) within the neutrophils from Cl^−^ and H_2_O_2_^31^. The formation of HOCl may further contribute to the consumption of GSH owing to the ability of this antioxidant molecule to interact with it^32^. Therefore, MPO not only amplified the inflammatory response within the hepatic tissue but also increased oxidative stress. This study showed that the animals administered olive oil had normal prooxidant inflammatory markers. However, the treatment of CCl_4_-exposed animals with VVF1 considerably decreased all of the studied inflammatory mediators relative to the CCl_4_ group. These outcomes were in line with Aouey *et al*.^33^ and may be attributed to the anti-inflammatory potential of its active ingredients, including ferulic, caffeic, p-coumaric, salicylic, and ellagic acids^24^ as well as flavonoids^33^. The efficiency of VVF1 was not only related to the presence of these polyphenols but also the synergistic effect between them. Hence, the synergistic anti-inflammatory activities of certain phenolics such as resveratrol and quercetin^34^ as well as phenolic-containing extracts have been reported before^35,36^. Moreover, the current research has confirmed the anti-inflammatory action of SM, which has been reported earlier^37^ and has shown a lower anti-inflammatory effect than our VVF1. These results proved and clarified the potent anti-inflammatory activity of this grape fraction.

Induction of both oxidative stress and inflammation in hepatic tissue by CCl_4_ was considered to be the driving force of necroptosis. This damage effect is a form of necrosis that mediated via death receptors such as TNF, Fas, and TNF-related apoptosis-inducing ligand (TRAIL). This process has happened following the activation of receptor-interacting protein kinase 3 (RIPK3) and MLKL, and the inhibition of caspase 8, which converts extrinsic apoptosis to necrosis. Activation of RIPK3 phosphorylates MLKL leading to its translocation into the plasma membrane inner leaflet, causing perforation of membrane and disruption of the cell integrity. MLKL is significant for the induction of necroptosis and is the one that decides whether the cell is undergoing necroptosis or apoptosis^38^. In the current study, the injection of CCl_4_ led to a dramatic increase in the hepatic level of MLKL, which confirms the induction of necroptosis in rat liver tissue. In addition, certain molecules released from necroptotic cells can trigger activation of hepatic stellate cells (HSCs)^39^, which play a key role in liver fibrosis after activation by TGF-β1 and TNF-α^40^. There are two essential markers (COL1A1 and TGF-β1) that imply HSC activation. After activation, they secrete collagen type I with other mediators to promote fibrogenesis^40^. Subsequently, the gene expression of both COL1A1 and TGF-β1 was significantly upregulated (Fig. 5B). Therefore, the injection of CCl_4_ to rats in this study induced necroptosis and activation of HSC to promote fibrogenesis. These results were in harmony with our histopathological outcomes that revealed the presence of hepatic necrosis and steatosis with inflammation (steatohepatitis) and deposition of collagen fibers. Moreover, the serum profile of these rats showed a significant elevation of cholesterol level, which could lead to the accumulation of the lipid droplets in the liver. These results were in accordance with the previous studies^41,42^. The ALT and AST activities showed no significant change compared to the control rats in addition to the dramatic depletion of albumin level. ALT and AST leaked and raised in serum after hepatocyte death then their levels dropped and returned to normal after a few days. However, albumin has a longer half-life in serum, coupled with the capacity of the liver to synthesize it, so its concentration shifts slowly^43^. Therefore, the normal activities of these transaminases with depletion of albumin indicate liver damage and injury in the CCl_4_ group. The administration of olive oil to rats in the V group did not significantly alter either the MLKL level or the studied pro-fibrotic mediators and only congestion and dilated sinusoids in its hepatic histopathological assessment. Thus, the main toxicity in CCl_4_-injected rats is related to CCl_4_ itself, not to olive oil.

The treatment with VVF1 massively reduced hepatic MLKL relative to rats in the CCl_4_ group. This was probably due to its phenolic-related antioxidant and anti-inflammatory activities. As VVF1 can diminish hepatic ROS, which plays a key role in the induction of TNF-mediated hepatic necroptosis thus CCl_4_-induced hepatic cell death, via elevation of MLKL, can be inhibited. No prior study has investigated the influence of VV on MLKL during hepatic necroptosis, so our research is the first to elucidate this point. However, few studies have investigated the influence of phenolic compounds on necroptosis. Recently, the protective effect of certain phenolic-containing extract against necroptosis was explored^44^. In contrast, gallic acid proved its efficiency in the induction of necroptosis-dependent death in the activating HSC and was considered as a new therapeutic strategy for the avoidance of hepatic fibrosis^45^. Therefore, phenolic compounds can perform dual actions during hepatotoxicity by stimulating the necroptosis, especially, in the activated HSCs, thereby halting fibrogenesis and preventing death for other hepatic cells. Subsequently, the phenolic compounds in VVF1 assumed inhibition of hepatic necroptosis and eliminated the activated HSCs by decreased MLKL level and TGF-β1 expression and, in turn, decreased the gene expression of COL1A1. These results were confirmed by the histopathological findings and the serum profile of liver function. The present research also disclosed the enhancing ability of SM for hepatic necroptosis, the depletion of pro-fibrotic mediators, and the improvement of the serum profile that was less potent than VVF1. In addition, the administration of SM or VVF1 without CCl_4_ had no toxicity on the liver.

## Conclusions

The highest polyphenol-enriched fraction of the seedless black VV fruit (VVF1) exhibited the strongest antioxidant-dependent anti-necroptotic impact against CCl_4_-exposed hepatocytes. Furthermore, this study declared, for the first time, the synergistic antioxidant and anti-necroptosis effectiveness of VVF1’s constitutes. Based on our best knowledge, no previous studies reported the *in vivo* efficacy of VVF1 to suppress necroptosis and fibrosis-mediated hepatic damage by the normalization of cellular redox status as well as lowering the pro-necroptotic protein (MLKL), -inflammatory and -fibrotic mediators (TGF-β1 and COL1A1). Additionally, VVF1 has been able to improve liver architecture (no necrosis, fibrosis, steatosis, and inflammation in contrast to the untreated CCl_4_ group) and functions. Moreover, these above-mentioned investigations revealed that VVF1 possessed higher anti-hepatotoxicity potentials than the standard SM drug and thus VVF1 considered as a new, effective natural anti-hepatotoxic agent for targeting necroptotic and profibrotic mediators.

## Materials and Methods

### Chemicals

4-Hydroxycinnamic acid (4-HCA), rutin (RU), α, α-diphenyl-β-picrylhydrazyl (DPPH), CCl_4_, Collagenase I, nitroblue tetrazolium (NBT), 2′,7′- dihydrodichlorofluorescein diacetate (DCFH-DA) probe, ethidium bromide (EB), acridine orange (AO), NaCN, riboflavin, thiobarbituric acid (TBA), tetramethoxypropane (TMP), reduced glutathione (GSH), annexin V, propidium iodide (PI), 3-(4,5-dimethylthiazol-2yl-)-2,5-diphenyl tetrazolium bromide (MTT), streptavidin-fluorescein, butylated hydroxytoluene (BHT), 2,2-azino-bis(3-ethylbenzthiazoline-6-sulfonic acid (ABTS), 5, 5′-dithio bis2- nitrobenzoic acid, and o-dianisidine dihydrochloride (ODD) were obtained from Sigma-Aldrich (St. Louis, MO, USA). Silymarin (SM) capsules were purchased from SEDICO Pharmaceuticals Company, Egypt. Each capsule contains SM 70% (200 mg), acetylcysteine (200 mg), vitamin E (5 IU), vitamin A (300 IU), vitamin C (30 mg), selenium (18.3 mg), and zinc (3.65 mg). Roswell Park Memorial Institute (RPMI)-1640 medium, William’s E medium and fetal bovine serum (FBS) were obtained from Lonza (USA). Gene JET RNA purification kit, cDNA synthesis kit, and 2X SYBR green master mix kit were supplied from Thermo Fisher Scientific, USA. MLKL and resveratrol ELISA kits were obtained from Cloud-clone Corp, USA and GmbH, Aachen, Germany, respectively. ALT and AST, albumin, and cholesterol kits were purchased from Biosystem, Spain. Primers were purchased from Bioneer, Korea. Other chemicals were obtained with a high grade.

### Animals

Fifty-nine male Albino rats were purchased from MISR University for Science and Technology with animal welfare (assurance number: A5865-01). Rats were acclimatized under the conventional conditions of about 30 °C with a 12-hour light-dark cycle for two weeks. During this period, animals allowed free access to tap water and a standard commercial diet. All relevant international and/or institutional recommendations for using animals were followed. The animal experiments follow the Research Ethical Committee that published by the National Health and Medical Research Council policies and the Ministry of Health and Population, High Committee of Medical Specialties, Egypt. The present study was approved by the General Authority of the City of Scientific Research and Technological Applications-Ministry of Scientific Research, Egypt.

### Plant material and preparation of the phenolic fractions

The VV (NCBI:txid29760) was imported from Lebanon and used for the preparation of three phenolic fractions. The black color fruit (seedless pulp with the skin) was ground using an electric grinder, then lyophilized (Telstar, Terrassa, Spain) to obtain the powdered VVCE (yield 14.020 ± 0.140 g/100 g grape). Then 50 g of VVCE was extracted twice with 70% ethanol (500 mL for each) using reflux for an hour at 50 °C. After filtration, the obtained extract (VVF1) was distilled to remove ethanol and then cooled for 12 h in a refrigerator (4 °C) for precipitation of the ethanol-soluble components. The precipitate (resveratrol-rich fraction, VVF2) was separated from the filtrate (water-soluble components, VVF3) by centrifugation for 10 min at 4000 rpm. The obtained fractions (VVF1, VVF2, and VVF3) were freeze-dried and stored at −20 °C until used.

### Spectrophotometric and chromatographic analysis of the VV polyphenols

The total phenolics and flavonoids in the crude extract (VVCE) and the prepared fractions (VVF1, VVF2, and VVF3) were determined spectrophotometrically. Total phenolics were quantified by the Folin-Ciocalteau method using 4-HCA calibration curve^46^. Total flavonoids were measured at 510 nm after mixing each fraction with 5% sodium nitrite and 10% AlCl_3_ and the concentration was calculated using RU standard curve^47^.

Twenty microliters of VVF1 were separated on the Zorbax Eclipse plusC18 column (100 mm × 4.6 mm Agilent Technologies, Palo Alto, CA, USA). The separation was achieved at 284 nm with a flow rate of 0.75 mL/min using a ternary linear elution gradient and a mobile phase of 0.2% H_3_PO_4_, methanol, and acetonitrile. Under similar chromatographic conditions, pure phenolic standards were run to match the retention items^48^.

The content of the resveratrol was determined using a specific ELISA kit. The kit depends on a competitive inhibition enzyme immunoassay technique using resveratrol-specific monoclonal antibody and avidin conjugated Horseradish Peroxidase.

### Antioxidant activities

The total antioxidant capacity (TAC), β-carotene-linoleate bleaching assay, and antiradical activities (anti-DPPH, superoxide, and hydroxyl radicals) of the crude and prepared phenolic fractions (VVCE, VVF1, VVF2, and VVF3) were tested. The value of IC_50_ (50% inhibitory concentration) for each studied radical and the β-carotene-linoleate bleaching was estimated by the GraphPad Instat software version 3.

The TAC of the studied fractions was determined using a mixture of ammonium molybdate (4 mM), sodium phosphate (28 mM), and H_2_SO_4_ (0.6 M). After 90 min at 95 °C, the reducing ability of the studied fractions to phosphomolybdate was read at 695 nm using Asc standard curve^49^.

The DPPH scavenging activity was evaluated following the modified standard method of Blois^50^ by incubating serial concentrations of VV crude or fractions with freshly prepared 0.004% DPPH (in methanol) at room temperature for 30 min. Then the absorbance of the non-scavenged DPPH was measured at 490 nm. Regarding, the hydroxyl radical scavenging activity was assessed at 510 nm after 60 min incubation of serial dilutions of samples with 9 mmol/L of salicylic acid, FeSO_4_ and H_2_O_2_, at 37 °C^51^. The method of McCord and Fridovich^52^ was used to determine the scavenging activity of the studied fractions to superoxide anion radical. In this method, serial concentrations of VV or each fraction were incubated with a reaction mixture containing 0.1 M EDTA, 1.5 mM NBT, 0.0015% NaCN, 0.12 mM riboflavin, and 67 mM phosphate buffer (pH 7.8) for 15 min. The absorbance was then recorded at 530 nm.

The β-carotene-linoleate bleaching method measured the anti-lipid peroxidation effect of the samples using β-carotene, linoleic acid, and Tween-80 emulsion^53^. The decrease in the bleaching rate of β-carotene which reflects the ability of each fraction to scavenge the radicals generated from linoleic acid oxidation was recorded. The absorbance (a, b) was read at 490 nm instantly and after 180 min (t), respectively. Then the value of the degradation rate (DR) of each fraction, standard (BHT), and control (without fraction) was calculated using the equation: [DR = ln (a/b) × (1/t)]. The antioxidant ability was determined as % of inhibition using the formula: Antioxidant activity (%) = (DR_control_ − DR_fraction_/DR_control_) × 100.

### *In vitro* evaluation of the most effective anti-hepatotoxic VV fractions

#### Assessment of the safe concentrations of VV fractions on the isolated hepatocytes

The liver of three male Albino rats (weighing 30–35 g, 2 weeks age) was used to isolate the hepatocytes according to the method of Whitehead and Robinson with some modifications^54^. After hepatocyte isolation using collagenase I and cultivation in William’s E medium supplemented with FBS (10%), the cytotoxicity of the phenolic fractions on the hepatocytes was tested using MTT assay^55^. Briefly, the isolated hepatocytes were seeded in 96-well cell culture plate and treated individually with serial concentrations of VVF1, VVF2, VVF3 as well as the standard drug for hepatotoxicity (SM). Also, the untreated cells were considered as a negative control sample. The plates were incubated for 72 h at 5% CO_2,_ 37 °C and 90% relative humidity in the CO_2_ incubator. Then MTT (5 mg/mL) was added to each well and incubated for an additional 4 h. At the end of the incubation period, MTT was replaced by 150 µl of DMSO and the absorbance was read at 570 nm using an ELISA reader (BMG LabTech, Germany). Safe concentration at 100% cell viability (EC_100_) was calculated using GraphPad InStat software.

#### *In vitro* induction of hepatotoxicity and treatment procedure

The hepatotoxicity was induced in the isolated rat hepatocytes by incubation with 0.13 mM CCl_4_ using the previous method of Abu-Serie & habashy^56^. After 36 h, cells were incubated with serial dilutions of safe concentration (EC_100_) of VV fractions and SM for 72 h in 5% CO_2_ incubator at 37 °C. The CCl_4_-exposed cells without any further treatments and the normal untreated hepatocytes were used as positive and negative control cells, respectively. Then the cell viability after each treatment compared with the control cells was assessed using MTT as indicated above^55^. The theoretical effective doses of each VV fraction and SM that terminated CCl_4_-induced hepatotoxicity by 100% (ED_100_) and 50% (ED_50_) were calculated by the GraphPad Instat software version 3. The further analyses of the VV fractions and SM effectiveness on the CCl_4_-induced hepatotoxicity were performed at the theoretical ED_100_ value for each sample.

#### Morphological examination of the CCl_4_-damaged hepatocytes after VV treatment

Before and after the treatment of the CCl_4_-exposed hepatocytes with the VV fractions and SM, cells were investigated morphologically using a phase-contrast microscope (Olympus, Japan). In addition, EB and AO dyes (100 µg/mL for each) were used for staining the cells to examine the influence of the VV fractions and SM on CCl_4_-induced hepatocyte death. Then the stained hepatocytes were visualized under the fluorescent phase-contrast microscope (Olympus, Japan).

#### Flow cytometric analysis of necroptotic hepatocytes and intracellular ROS quantification

The quantification of necroptosis was determined by the flow cytometer using annexin V/PI stain. The untreated and treated hepatocytes were trypsinized and incubated with isothiocyanate (FITC)-labelled annexin V/PI for 15 min. The choice of the most effective anti-hepatotoxic VV fraction was established by quantification of the annexin/PI-stained population in the studied cells. This was done by the flow cytometer using the phycoerythrin emission signal detector (FL2) against the FITC signal detector (FL1).

The DCFH-DA (5 μM) probe was used for the quantification of the intracellular ROS level^57^. The untreated and treated hepatocytes were incubated in the dark with the fluorescent probe for 30 min at 37 °C. Then cells were trypsinized and suspended in phosphate-buffered saline (PBS) to analyze the fluorescence intensity by flow cytometer (Partec, Germany). The analysis was done at an excitation and an emission wavelength of 488 and 530 nm, respectively.

#### Combination index (CI) analysis

The combination of VVF2 phenolics with VVF3 phenolics in VVF1 may or may not (additive effect) confer higher (synergistic) or lower (antagonistic) antioxidant and anti-hepatotoxic activity for VVF1. The probable new antioxidant effect can be evaluated using the effect “Fa”-CI and isobologram plots. In addition, the CI values at Fa 0.5 (50% inhibition), Fa 0.75 (75% inhibition), and Fa 0.9 (90% inhibition) were calculated. Both the plots and the CI values were generated by the current software, CompuSyn. However, the anti-hepatotoxic (*in vitro*) activity (% necroptotic cells and the intracellular ROS) of VVF1 was detected using the CI values that were calculated by dividing the expectable value by the observed value. The CI value may be ˂ 1 (synergistic effect), equal to 1 (additive effect), or ˃ 1 (antagonistic effect)^48^.

### *In vivo* evaluation for the anti-hepatotoxic effect of the most effective VV fraction

#### Experimental scheme

Fifty-six rats (weighing 140–200 g, 6 weeks’ age) were divided randomly into seven groups (eight animals in each). The treatment of animals in each experimental group is illustrated in Fig. 1. Briefly, hepatotoxicity was induced in rats by intraperitoneal (i.p.) injection of 1 ml 50% CCl_4_ in olive oil/kg b.w./twice/week for 3 weeks^58^. After these 3 weeks, CCl_4_-VVF1 and CCl_4_-SM rat groups were orally injected with 1.5 g VVF1 and 50 mg SM/kg b.w., respectively, using gavage, daily for 10 days. At the end of the experimental period (day 30), rats were anesthetized and dissected then blood (by cardiac puncture) and liver tissues were collected immediately. The heparinized blood was centrifuged at 6000 rpm (15 min) to separate plasma for assessment of the liver function markers, cholesterol, and TAC. Liver tissues were washed with cold saline (0.9% NaCl), weighted and small portions were fixed in 10% formalin for histopathological investigation. The remaining liver tissue was kept at −80 °C until used in the biochemical and molecular analyses.

#### Assessment of necroptotic and fibrotic mediators in liver tissues

The necroptotic mediators (oxidative stress and necroinflammation), as well as the fibrotic mediators (TGF-β and COL1A1), were assessed at mRNA and protein levels in rat livers to evaluate the anti-hepatotoxic role of VVF1 *in vivo*.

#### Biochemical assessment of hepatic oxidative stress and inflammation-dependent necroptotic mediators

The oxidative stress (cellular redox state disruption)-mediated inflammation in the liver homogenate was assessed by determination of the intracellular ROS, NO, TAC, lipid peroxidation, MPO and the antioxidant indices levels. The homogenates were prepared by homogenizing the liver of each rat in each experimental group in fresh cold PBS (1:10 w/v) then centrifuged at 6000 rpm (4 °C) for 30 min and the clear supernatants were used for the analyses.

The ROS level was assessed using the extremely sensitive DCFH-DA (5 μM) fluorescent probe. In brief, the diluted clear homogenate (2-fold dilution with PBS) of each sample was mixed with an equal volume of diluted DCFH-DA (1000-fold dilution with 10 µM dimethyl sulfoxide). Then the reaction mixtures were incubated, in dark, for 5 min at 37 °C and finally, the fluorescence intensity was measured at 485 (excitation) and 520 nm (emission) to calculate the ROS level using H_2_O_2_ standard curve^59^.

NO was determined as nitrite using a Griess reaction that produced colored azo dye with a maximum absorbance at 490 nm^60^. The lipid peroxidation level was examined by TBA reactive substances (TBARS) colorimetric method^61^ using TMP calibration curve.

Myeloperoxidase activity was examined colorimetrically using ODD (16.7 mg%) and H_2_O_2_ (1.2%) as described previously^62^. The enzyme activity was measured as IU/mg protein, where one IU is defined as the amount of the enzyme that able to degrade 1 μmoL of H_2_O_2_/min at 25 °C.

The antioxidant indices comprising the activities of SOD and GPX were assessed using the pyrogallol autooxidation assay^63^ and Rotruck method^64^, respectively. The specific activities of SOD and GPX were determined by dividing the activity of each enzyme by the protein content in the homogenate. The protein level was assessed using biuret method followed the manual protocol of the specific kit. In addition, the level of nonenzymatic antioxidant (GSH) was determined using Ellman’s reagent (5, 5′-dithio bis2- nitrobenzoic acid) and its concentration was calculated from the GSH calibration curve^65^.

The level of MLKL (a marker of the necrosis or inflammatory cell death) was determined using the specific ELISA kit by following the manufacturer’s instructions.

#### Molecular assessment of necroptotic and pro-fibrotic mediators

The markers of necroinflammation (NF-кB, COX-2, TNF-α, and iNOS) and fibrosis (COL1A1 and TGF-β1) were assessed at mRNA in rat livers. The liver of rats in each experimental group was homogenized in lysis buffer containing β-mercaptoethanol and then centrifuged at 14,000 rpm for 5 min. The total RNA in the separated supernatant was extracted using Gene JET RNA Purification Kit and quantified then the cDNA was synthesized using the cDNA Synthesis Kit. The gene expression levels of target genes relative to glyceraldehyde-3-phosphate dehydrogenase (GAPDH, housekeeping gene) were measured by real-time PCR using SYBR green master mix and specific primers. The following primers were used: ***NF-кB***, forward 5′-TGCTAATGGTGGACCGCAA-3, reverse: 5′-CACTGCTTCCCGAATGTCTGA-3′; ***COX-2***, forward: 5′-CCCATGTCAAAACCGTGGTG-3′, reverse: 5′-CTTGTCAGGAATCTCGGCGT-3′; ***TNF-α***, forward:5′-GCCCAGACCCTCACACTC-3′; reverse: 5′-CCACTCCAGCTGCTCCTCT-3′; ***iNOS***, forward: 5′-ACCATGGAGCATCCCAAGTA-3′, reverse: 5′-CAGCGCATACCACTTCAGC-3′; ***COL1A1***, forward: 5′- CATGTTCAGCTTTGTGGACCT-3′, reverse: 5′-GCAGCTGACTTCAGGGATGT-3′; and ***TGF-β1***, forward: 5′- TGCTAATGGTGGACCGCAA-3′, reverse: 5′- CACTGCTTCCCGAATGTCTGA-3′. The fold expression of the target genes was calculated via the comparative Ct method (threshold cycle number at cross-point between threshold and amplification plot).

#### Histopathological examination

After fixation, liver tissue specimens were processed by following the routine protocol for histopathological investigation. Hence, the samples were embedded in paraffin wax and 5 µm thickness slices were cut and stained with hematoxylin and eosin. The pathological features of liver tissues in all the studied groups were visualized by the phase-contrast microscope then high-resolution images were captured at 200x magnification.

#### Plasma analyses

The liver function markers, including ALT, AST, albumin as well as cholesterol level were determined spectrophotometrically in plasma samples of rats in all the studied groups using the specific kits.

#### Statistical analysis

The data are expressed as mean ± SE and the p-value < 0.05 is considered significant. The difference between the mean values of the studied groups was evaluated by one-way analysis of variance (ANOVA) by Tukey’s test. Before applying this parametric test, all data were checked for their normal distribution (skewness 0–0.868). The analysis was performed for seven rats using SPSS software version 16. The IC_50_, EC_100_, ED_50_, and ED_100_ values were calculated by GraphPad Instate software version 3. In addition, the CompuSyn software (ComboSyn, Inc, Paramus, NJ) accomplished the CI values, Fa-CI plots and isobologram plots for the *in vitro* antioxidant experiments.

## Supplementary information

Supplementary Figure 1.

Supplementary Figure 2.

## Data Availability

All data produced during this study is included in this published article.

## References

[1] Xu C, Li CYT, Kong ANT (2005). Induction of phase I, II and III drug metabolism/transport by xenobiotics. Arch. Pharm. Res..

[2] Weber LWD, Boll M, Stampfl A (2003). Hepatotoxicity and mechanism of action of haloalkanes: Carbon tetrachloride as a toxicological model. Crit. Rev. Toxicol..

[3] Mitra A (2014). IL-30 (IL27p28) attenuates liver fibrosis through inducing NKG2D-rae1 interaction between NKT and activated hepatic stellate cells in mice. Hepatol..

[4] Christofferson DE, Yuan J (2010). Necroptosis as an alternative form of programmed cell death. Curr. Opin. Cell Biol..

[5] This P, Lacombe T, Thomas MR (2006). Historical origins and genetic diversity of wine grapes. Trends Genet..

[6] Tabeshpour J, Mehri S, Shaebani Behbahani F, Hosseinzadeh H (2018). Protective effects of *Vitis vinifera* (grapes) and one of its biologically active constituents, resveratrol, against natural and chemical toxicities: A comprehensive review. Phytother. Res..

[7] Hasona, N. A., Alrashidi, A. A., Aldugieman, T. Z., Alshdokhi, A. M. & Ahmed, M. Q. *Vitis vinifera* extract ameliorate hepatic and renal dysfunction induced by dexamethasone in albino rats. *Toxics* **5** (2017). 10.3390/toxics5020011 PMC5606666 29051443

[8] Nassiri-Asl, M. & Hosseinzadeh, H. Review of the pharmacological effects of *Vitis vinifera* (grape) and its bioactive constituents: An update. *Phytother Res*. 1392–1403 (2016). 10.1002/ptr.5644 27196869

[9] Sharma SK, Suman, Vasudeva N (2012). Hepatoprotective activity of *Vitis vinifera* root extract against carbon tetrachloride-induced liver damage in rats. Acta Pol. Pharm. - Drug. Res..

[10] Orhan DD, Orhan N, Ergun E, Ergun F (2007). Hepatoprotective effect of *Vitis vinifera* L. leaves on carbon tetrachloride-induced acute liver damage in rats. J. Ethnopharmacol..

[11] Ragab GMA (2013). Grape (*vitis vinifera*) seed extract inhibits the cytotoxicity and oxidative stress in liver of rats treated with carbon tetrachloride. Glob. J. Pharmacol..

[12] Pirinccioglu M (2012). Protective effect of Öküzgözü (*Vitis vinifera* L. cv.) grape juice against carbon tetrachloride induced oxidative stress in rats. Food Funct..

[13] Maqsood S, Benjakul S, Abushelaibi A, Alam A (2014). Phenolic compounds and plant phenolic extracts as natural antioxidants in prevention of lipid oxidation in seafood: A detailed review. Comp. Rev. Food Sci. Food Saf..

[14] Hugo PC (2012). Antioxidant interactions between major phenolic compounds found in ‘Ataulfo’ mango pulp: Chlorogenic, gallic, protocatechuic and vanillic acids. Molecules.

[15] Sonam KS, Guleria S (2017). Synergistic Antioxidant Activity of Natural Products. Ann. Pharmacol. Pharm..

[16] Hajimehdipoor H, Shahrestani R, Shekarchi M (2014). Investigating the synergistic antioxidant effects of some flavonoid and phenolic compounds. Res. J. Pharmacogn..

[17] Bisht S (2011). A polymeric nanoparticle formulation of curcumin (NanoCurc^TM^) ameliorates CCl_4_-induced hepatic injury and fibrosis through reduction of pro-inflammatory cytokines and stellate cell activation. Lab. Invest..

[18] Xiao J (2012). Lycium barbarum polysaccharides protect mice liver from carbon tetrachloride-induced oxidative stress and necroinflammation. J. Ethnopharmacol..

[19] Roesler R (2011). Effect of extracts from araticum (*Annona crassiflora*) on CCl_4_-induced liver damage in rats. Food Sci. Technol..

[20] Kunwar A, Priyadarsini K (2011). Free radicals, oxidative stress and importance of antioxidants in human health. J. Med. Allied Sci..

[21] Adewale OB, Adekeye AO, Akintayo CO, Onikanni A (2014). S. S. Carbon tetrachloride (CCl_4_)-induced hepatic damage in experimental Sprague Dawley rats: Antioxidant potential of *Xylopia aethiopica*. J. Phytopharmacol.

[22] Kahkeshani N (2019). Pharmacological effects of gallic acid in health and diseases: A mechanistic review. Iran. basic. med. sci..

[23] Vinothiya K, Ashokkumar N (2017). Modulatory effect of vanillic acid on antioxidant status in high fat diet-induced changes in diabetic hypertensive rats. Biomed. Pharmacother..

[24] Szwajgier, D., Borowiec, K. & Pustelniak, K. The neuroprotective effects of phenolic acids: Molecular mechanism of action. *Nutrients* **9** (2017). 10.3390/nu9050477 PMC5452207 28489058

[25] Moskaug, J. O., Carlsen, H., Myhrstad, M. C. W. & Blomhoff, R. Polyphenols and glutathione synthesis regulation. *Am J Clin Nutr* **81** (2005). 10.1093/ajcn/81.1.277S 15640491

[26] Surai PF (2015). Silymarin as a natural antioxidant: An overview of the current evidence and perspectives. Antioxid..

[27] Raj S, Gothandam KM (2014). Hepatoprotective effect of polyphenols rich methanolic extract of *Amorphophallus commutatus* var. wayanadensis against CCl_4_ induced hepatic injury in swiss albino mice. Food Chem. Toxicol..

[28] Shaker E, Mahmoud H, Mnaa S (2010). Silymarin, the antioxidant component and *Silybum marianum* extracts prevent liver damage. Food Chem. Toxicol..

[29] Morgan, M. J. & Liu, Z. G. Crosstalk of reactive oxygen species and NF-κB signaling. *Cell Res*. 103–115 (2011). 10.1038/cr.2010.178 PMC3193400 21187859

[30] Zhang R (2002). Myeloperoxidase functions as a major enzymatic catalyst for initiation of lipid peroxidation at sites of inflammation. J. Biol. Chem..

[31] Kettle AJ, Winterbourn CC (1990). Superoxide enhances hypochlorous acid production by stimulated human neutrophils. Biochim. Biophys. Acta.

[32] Winterbourn CC (1985). Comparative reactivities of various biological compounds with myeloperoxidase-hydrogen peroxide-chloride, and similarity of oxidant to hypochlorite. Biochim. Biophys. Acta.

[33] Aouey B, Samet AM, Fetoui H, Simmonds MSJ, Bouaziz M (2016). Anti-oxidant, anti-inflammatory, analgesic and antipyretic activities of grapevine leaf extract (*Vitis vinifera*) in mice and identification of its active constituents by LC–MS/MS analyses. Biomed. Pharmacother..

[34] Zhao L (2017). Combination treatment with quercetin and resveratrol attenuates high fat diet-induced obesity and associated inflammation in rats via the AMPKα1/SIRT1 signaling pathway. Exper Ther. Med..

[35] Habashy, N. H., Abu Serie, M. M., Attia, W. E. & Abdelgaleil, S. A. M. Chemical characterization, antioxidant and anti-inflammatory properties of Greek *Thymus vulgaris* extracts and their possible synergism with Egyptian *Chlorella vulgaris*. *J Funct Foods* (2018).

[36] Abu-Serie, M. M., Habashy, N. H. & Attia, W. E. *In vitro* evaluation of the synergistic antioxidant and anti-inflammatory activities of the combined extracts from Malaysian *Ganoderma lucidum* and Egyptian *Chlorella vulgaris*. *BMC Complement Alterne Med* (2018). 10.1186/s12906-018-2218-5 PMC5946467 29747629

[37] Abdel-Moneim, A. M., Al-Kahtani, M. A., El-Kersh, M. A. & Al-Omair, M. A. Free radical-scavenging, anti-inflammatory/anti-fibrotic and hepatoprotective actions of taurine and silymarin against CCl_4_ induced rat liver damage. *PLoS ONE* **10** (2015). 10.1371/journal.pone.0144509 PMC4676695 26659465

[38] Dhuriya, Y. K. & Sharma, D. Necroptosis: A regulated inflammatory mode of cell death. *J Neuroinflammation* **15** (2018). 10.1186/s12974-018-1235-0 PMC6035417 29980212

[39] Luedde T, Kaplowitz N, Schwabe RF (2014). Cell death and cell death responses in liver disease: Mechanisms and clinical relevance. Gastroenterol..

[40] Reeves, H. L. & Friedman, S. L. Activation of hepatic stellate cells-a key issue in liver fibrosis. *Front biosci* **7** (2002). 10.2741/reeves 11897564

[41] Jeong WI (2005). Mild hepatic fibrosis in cholesterol and sodium cholate diet-fed rats. J. Vet. Med. Sci..

[42] Abdel-sttar AR, Khalaf MM, Aboyoussef AM, Abosaif AA (2017). Ameliorative effect of hesperidin on carbon tetrachloride induced liver fibrosis in rats. Inter. J. Pharm. Pharm Sci..

[43] Pradeep K, Mohan CVR, Gobianand K, Karthikeyan S (2007). Silymarin modulates the oxidant-antioxidant imbalance during diethylnitrosamine induced oxidative stress in rats. Eur. J. Pharmacol..

[44] Lee, Y. *et al*. Terminalia Chebula provides protection against dual modes of necroptotic and apoptotic cell death upon death receptor ligation. *Sci Rep* **6** (2016). 10.1038/srep25094 PMC4846877 27117478

[45] Chang Ya Ju, Hsu Shih Lan, Liu Yi Ting, Lin Yu Hsuan, Lin Ming Hui, Huang Shu Jung, Ho Ja-an Annie, Wu Li-Chen (2015). Gallic Acid Induces Necroptosis via TNF–α Signaling Pathway in Activated Hepatic Stellate Cells. PLOS ONE.

[46] Taga MS, Miller EE, Pratt DE (1984). Chia seeds as a source of natural lipid antioxidants. J. Am. Oil Chem. Soc..

[47] Zhishen J, Mengcheng T, Jianming W (1999). The determination of flavonoid contents in mulberry and their scavenging effects on superoxide radicals. Food Chem..

[48] Abu-Serie, M. M. & Habashy, N. H. The ameliorating effect of the combined extract from Greek *Thymus vulgaris* and bee’s honey on the hydrocortisone-induced osteoporosis in rat bone cells via modulating the bone turnover, oxidative stress, and inflammation. *RSC Advances* (2018). 10.1039/c8ra04370a PMC9084251 35542490

[49] Tyagi S, Singh A, Saxena A, Patel B (2010). *In vitro* Antioxidant Activity of Methanolic and aqueous extract of *Flacourtia indica Merr*. Am. J. Sci. Res..

[50] Blois MS (1958). Antioxidant determinations by the use of a stable free radical. Nat..

[51] Smirnoff N, Cumbes QJ (1989). Hydroxyl radical scavenging activity of compatible solutes. Phytochem..

[52] McCord JM, Fridovich I (1969). Superoxide dismutase. An enzymic function for erythrocuprein (hemocuprein). J. Biol. Chem..

[53] Barreira JCM, Ferreira ICFR, Oliveira MBPP, Pereira JA (2008). Antioxidant activities of the extracts from chestnut flower, leaf, skins and fruit. Food Chem..

[54] Whitehead, R. H. & Robinson, P. S. Establishment of conditionally immortalized epithelial cell lines from the intestinal tissue of adult normal and transgenic mice. *Am J Physiol Gastrointest Liver Physiol* **296**, G455–G460 (2009). 10.1152/ajpgi.90381.2008 PMC2660172 19109407

[55] Mosmann T (1983). Rapid colorimetric assay for cellular growth and survival: Application to proliferation and cytotoxicity assays. J. Immunol. Methods.

[56] Abu-Serie, M. M. & Habashy, N. H. Two purified proteins from royal jelly with *in vitro* dual anti-hepatic damage potency: Major royal jelly protein 2 and its novel isoform X1. *Inter J Biol Macromol* (2019). 10.1016/j.ijbiomac.2019.01.210 30711561

[57] Simizu S, Imoto M, Masuda N, Takada M, Umezawa K (1996). Involvement of hydrogen peroxide production in erbstatin-induced apoptosis in human small cell lung carcinoma cells. Cancer Res..

[58] Ikeda H, Watanabe N, Ishii I, Shimosawa T, Kume Y, Tomiya T, Inoue Y, Nishikawa T, Ohtomo N, Tanoue Y, Iitsuka S, Fujita R, Omata M, Chun J, Yatomi Y (2009). Sphingosine 1-phosphate regulates regeneration and fibrosis after liver injury via sphingosine 1-phosphate receptor 2. J. Lipid Res..

[59] Crow JP (1997). Dichlorodihydrofluorescein and dihydrorhodamine 123 are sensitive indicators of peroxynitrite *in vitro*: Implications for intracellular measurement of reactive nitrogen and oxygen species. Nitric Oxide.

[60] Marcocci L, Maguire JJ, Droy-Lefaix MT, Packer L (1994). The nitric oxide-scavenging properties of *Ginkgo biloba* extract EGb 761. Biochem. Biophys. Res. Commun..

[61] Ohkawa H, Ohishi N, Yagi K (1979). Assay for lipid peroxides in animal tissues by thiobarbituric acid reaction. Anal. Biochem..

[62] Kim JJ, Shajib S, Manocha MM, Khan WI (2012). Investigating intestinal inflammation in DSS-induced model of IBD. J. Vis. Exp..

[63] Marklund S, Marklund G (1974). Involvement of the superoxide anion radical in the autoxidation of pyrogallol and a convenient assay for superoxide dismutase. Eur. J. Biochem..

[64] Rotruck JT (1973). Selenium: Biochemical role as a component of glatathione peroxidase. Sci..

[65] Ellman GL (1959). Tissue sulfhydryl groups. Arch. Biochem. Biophys..

