# Supplementary material for: Vitis vinifera polyphenols from seedless black fruit act synergistically to suppress hepatotoxicity by targeting necroptosis and pro-fibrotic mediators
Source: Sci Rep. 2020 Feb 12;10:2452. doi: 10.1038/s41598-020-59489-z (PMC7016101; doi:10.1038/s41598-020-59489-z)
Supplement: Supplementary file 2 — Supplementary Figure 2. [file 41598_2020_59489_MOESM2_ESM.pdf]

Vitis vinifera polyphenols from seedless black fruit act synergistically to suppress hepatotoxicity by targeting necroptosis and pro-fibrotic mediators

Marwa M. Abu-Serie [a*], Noha H. Habashy[b].

[a]Department of Medical Biotechnology, Genetic Engineering, and Biotechnology Research Institute, City for Scientific Research and Technology Applications (SRTA-City), New Borg EL-Arab 21934, Alexandria, Egypt. **Phone:** +2034593422 **Fax:** +2034593407

[b]Biochemistry Department, Faculty of Science, Alexandria University, Alexandria 21511, Egypt.

[*]Correspondence: marwaelhedaia@gmail.com and noha.habashi@alexu.edu.eg

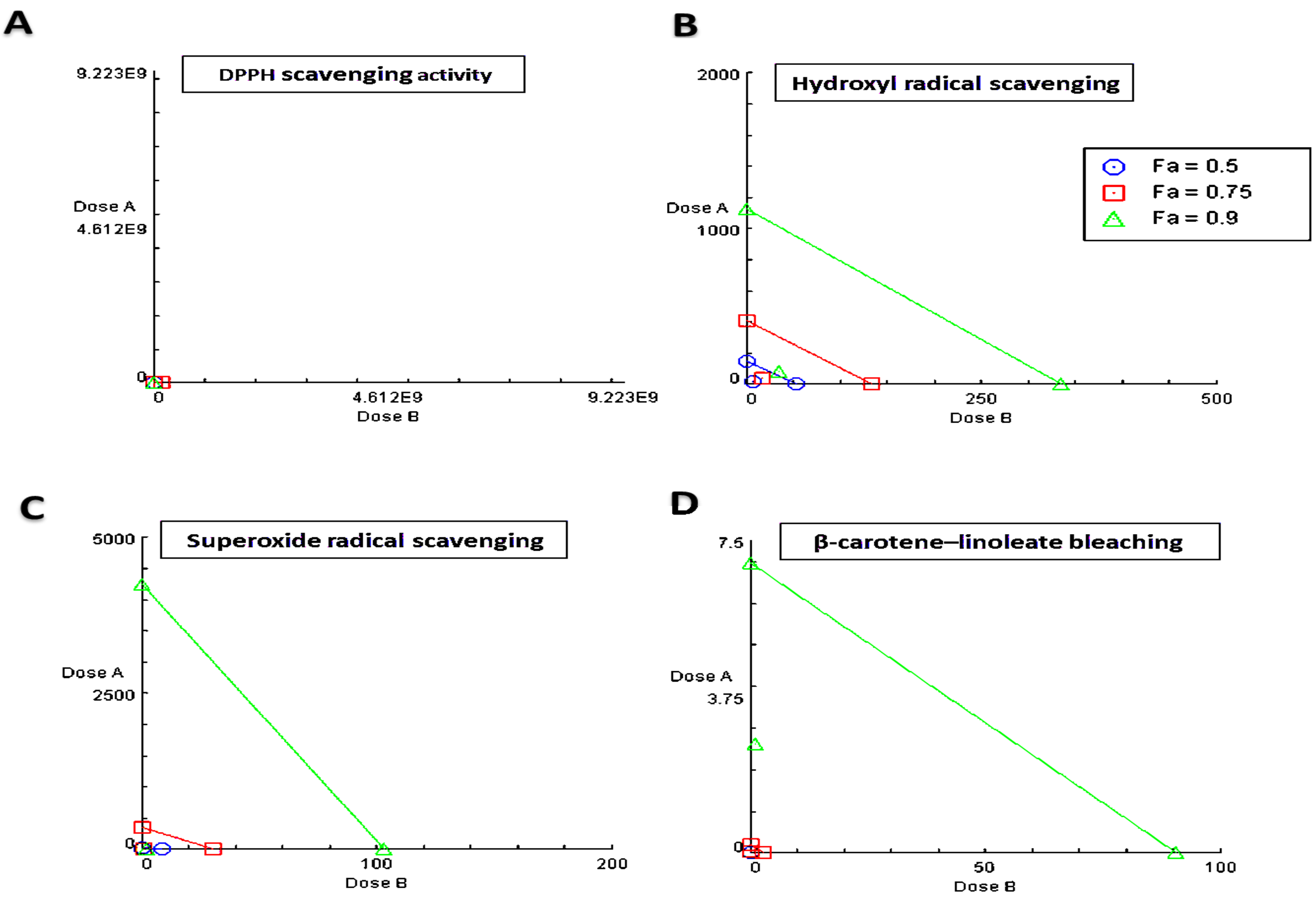

Supplementary Figure 2
